# U_S_3 Serine/Threonine Protein Kinase from MDV-1, MDV-2, and HVT Differentially Regulate Viral Gene Expression and Replication

**DOI:** 10.3390/microorganisms9040785

**Published:** 2021-04-09

**Authors:** Yifei Liao, Xin Fang, Mohammad AI-Mahmood, Qinglei Li, Blanca Lupiani, Sanjay M. Reddy

**Affiliations:** 1 Department of Veterinary Pathobiology, College of Veterinary Medicine & Biomedical Sciences, Texas A&M University, College Station, TX 77843, USA; mal-mahmood@cvm.tamu.edu (M.A.-M.); blupiani@tamu.edu (B.L.); 2 Department of Veterinary Integrative Biosciences, College of Veterinary Medicine & Biomedical Sciences, Texas A&M University, College Station, TX 77843, USA; xfang@cvm.tamu.edu (X.F.); QLi@cvm.tamu.edu (Q.L.)

**Keywords:** Marek’s disease virus, U_S_3, gene expression, replication, lymphocytes

## Abstract

*Gallid alphaherpesvirus* 2 (GaHV-2), commonly known as Marek’s disease virus type 1 (MDV-1), is an oncogenic avian alphaherpesvirus, and along with its close relatives—*Gallid alphaherpesvirus* 3 (GaHV-3) or MDV-2 and *Meleagrid alphaherpesvirus* 1 (MeHV-1) or turkey herpesvirus (HVT)—belongs to the *Mardivirus* genus. We and others previously showed that MDV-1 U_S_3 protein kinase plays an important role in viral replication and pathogenesis, which could be partially compensated by MDV-2 and HVT U_S_3. In this study, we further studied the differential roles of MDV-1, MDV-2 and HVT U_S_3 in regulating viral gene expression and replication. Our results showed that MDV-2 and HVT U_S_3 could differentially compensate MDV-1 U_S_3 regulation of viral gene expression in vitro. MDV-2 and HVT U_S_3 could also partially rescue the replication deficiency of MDV-1 U_S_3 null virus in the spleen and thymus, as determined by immunohistochemistry analysis of MDV-1 pp38 protein. Importantly, using immunohistochemistry and dual immunofluorescence assays, we found that MDV-2 U_S_3, but not HVT U_S_3, fully compensated MDV-1 U_S_3 regulation of MDV-1 replication in bursal B lymphocytes. In conclusion, our study provides the first comparative analysis of U_S_3 from MDV-1, MDV-2 and HVT in regulating viral gene expression in cell culture and MDV-1 replication in lymphocytes.

## 1. Introduction

Marek’s disease (MD) is a lymphoproliferative disease of chickens, caused by an oncogenic avian alphaherepsvirus, *Gallid alphaherpesvirus* 2 [GaHV-2], commonly known as Marek’s disease virus type 1 (MDV-1). Susceptible chickens become infected with MDV-1 through the respiratory route by inhaling dander shed by infected chickens. MDV-1 infection exhibits distinct lytic and latent phases where various immune cells are involved [1]. During the early stage of MDV-1 infection, it is widely accepted that the infected lung phagocytic cells, such as macrophage, transfer the virus to B lymphocytes, which further infect activated T lymphocytes [2]. However, a recent study, using a knockout chicken line where B lymphocytes maturation and antibody production were abrogated, showed that B lymphocytes are dispensable for MDV-1 replication and oncogenesis [3]. Therefore, the mechanisms of MDV-1 infection and its interplay with the host immune system remain to be explored.

U_S_3 is a conserved serine/threonine protein kinase encoded by alphaherpesviruses, which has been shown to be important for virus genome replication, nucleocapsid nuclear egress, apoptosis inhibition, transcription regulation, and immune evasion [4]. Alphaherpesvirus encoded U_S_3 protein kinase contains a conserved kinase activity domain that includes an ATP-binding site and a catalytic active site, which is essential for its kinase activity [4]. The substrates of herpes simplex virus type 1 (HSV-1) U_S_3 include viral proteins, such as glycoprotein B (gB), nuclear phosphoprotein U_L_31, and membrane phosphoprotein U_L_34, and cellular proteins, such as cAMP response element-binding protein (CREB), histone deacetylase 1 (HDAC1), HDAC2, p65, and programmed cell death protein 4 (PDCD4) [5,6,7,8,9,10]. U_S_3 of MDV-1 was first described in 1993 and was shown not to be essential for virus replication in vitro [11]. Later, Schumacher et al. demonstrated that MDV-1 U_S_3 shared the functions of other alphaherpesviruses-encoded U_S_3 orthologs in regulating virion morphogenesis, apoptosis and host cytoskeleton structure [12,13]. They also showed that U_S_3 interacts with phosphorylates MDV-1 pp38 [13], a viral protein important for MDV replication in B lymphocytes [14,15]. Recently, we identified more MDV-1 U_S_3 substrates, including MDV-1 Meq oncoprotein, cellular CREB and HDAC1 and 2, and characterized the role of MDV-1 U_S_3-mediated phosphorylation in regulating cellular and viral gene expression, protein interactions and virus replication [16,17]. In addition, we found that MDV-1 U_S_3 disrupts the promyelocytic leukemia protein nuclear bodies (PML-NBs) in a U_S_3 kinase activity and host proteasomal pathway-dependent manner similar to HSV-2 U_S_3 [18].

## 2. Materials and Methods

### 2.1. Viruses and Cell Culture

All viruses used in this study were generated previously using a very virulent plus strain, 686, of MDV-1 [16,17]. Chicken embryonic fibroblasts (CEF) were maintained at 37 °C in the presence of 5% CO_2_ in Leibowitz–McCoy (LM, 1:1) medium supplemented with 5% newborn calf serum.

### 2.2. Quantitative Polymerase Chain Reaction (qPCR) and Western Blot

CEF seeded on 60 mm dishes were infected with 300 plaque-forming units (PFU) of different viruses or remained uninfected. Seven days later, cells were harvested for RNA, DNA and protein isolation as described previously [16].

All qPCR were performed in a CFX96 Real time PCR Detection System using iTaq Universal SYBR Green Supermix, and the results were analyzed using the 2^−ΔΔCT^ method.

Proteins extracted from infected and uninfected CEF were subjected to sodium dodecyl sulphate-polyacrylamide gel electrophoresis (SDS-PAGE), followed by Western blot analysis with antibodies to MDV pp38 and chicken HSP90 protein. The density of each protein band was quantified using Image J software.

Western blot and qPCR were performed in two independent experiments and the statistical differences analyzed using *t*-test.

### 2.3. Immunohistochemistry (IHC) and Immunofluorescence Assay (IFA)

One-day-old specific-pathogen-free (SPF) chickens were inoculated subcutaneously with 2000 PFU of viruses or remained uninoculated as described previously [17].

*IHC of lymphoid organ sections*. At six days post-inoculation, lymphoid organs (spleen, thymus, and bursa) were collected from virus-inoculated or uninoculated (negative control) chickens, fixed in 10% neutral buffered formalin solution for 48 h and stored in 70% ethanol until used. Tissue sections were prepared and subjected to immunostaining with anti-MDV-1 pp38 monoclonal antibody and VECTASTAIN ABC kit according to the manufacturer’s instructions, using DAB (3,3’-diaminobenzidine) as the substrate. A representative image of three independent samples from each group is presented.

*IFA of bursa sections*. Bursa tissue sections, prepared from virus-inoculated or uninoculated (negative control) chickens, were subjected to IFA. After blocking with 5% bovine serum albumin (BSA), sections were incubated with mouse anti-pp38 antibody for 1 h at room temperature, followed by another hour incubation with Alexa Flour 488 conjugated goat anti-mouse antibody. After three washes with phosphate-buffered saline (PBS), sections were stained with Alexa Flour 647 conjugated mouse anti-chicken Bu-1 antibody (B lymphocyte marker) for 1 h at room temperature. 4′,6-diamidino-2-phenylindole (DAPI) was used to stain the cell nuclei. A representative image of three independent samples from each group is presented.

## 3. Results and Discussion

**Figure 1:**
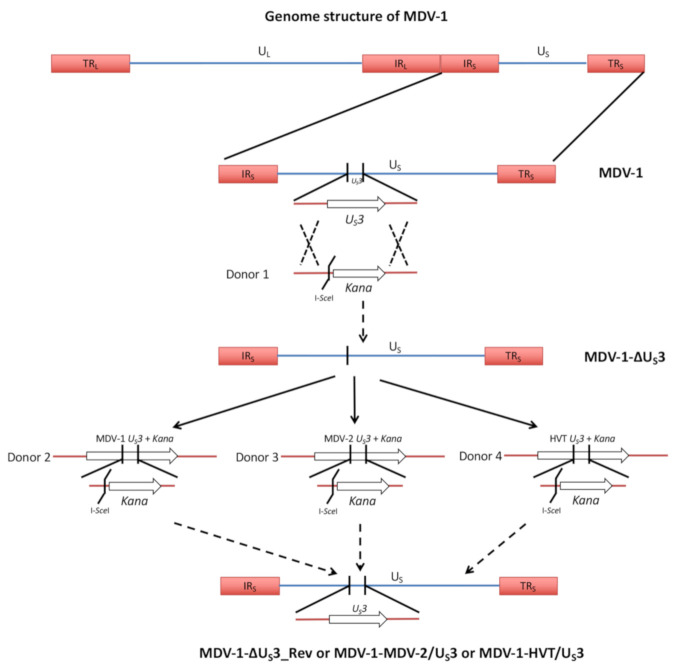
Construction of U_S_3 deletion, revertant and chimeric MDVs. MDV-1 genome consists of unique long and short regions (U_L_, U_S_) and inverted repeat long and short regions (TR_L_, IR_L_, TR_S_, IR_S_). U_S_3 was deleted from a bacterial artificial chromosome (BAC) containing the genome MDV-1, strain 686, using a two-step Red-mediated recombination method (MDV-1-ΔU_S_3 BAC). The U_S_3 deletion BAC was used as a backbone to construct MDV-1-ΔU_S_3_Rev (revertant), MDV-1-MDV-2/U_S_3 (chimeric MDV-1 expressing MDV-2 U_S_3), and MDV-1-HVT/U_S_3 (chimeric MDV-1 expressing HVT U_S_3) BACs. All BACs were transfected into chicken embryonic fibroblasts (CEF) to produce recombinant viruses.

**Figure 2:**
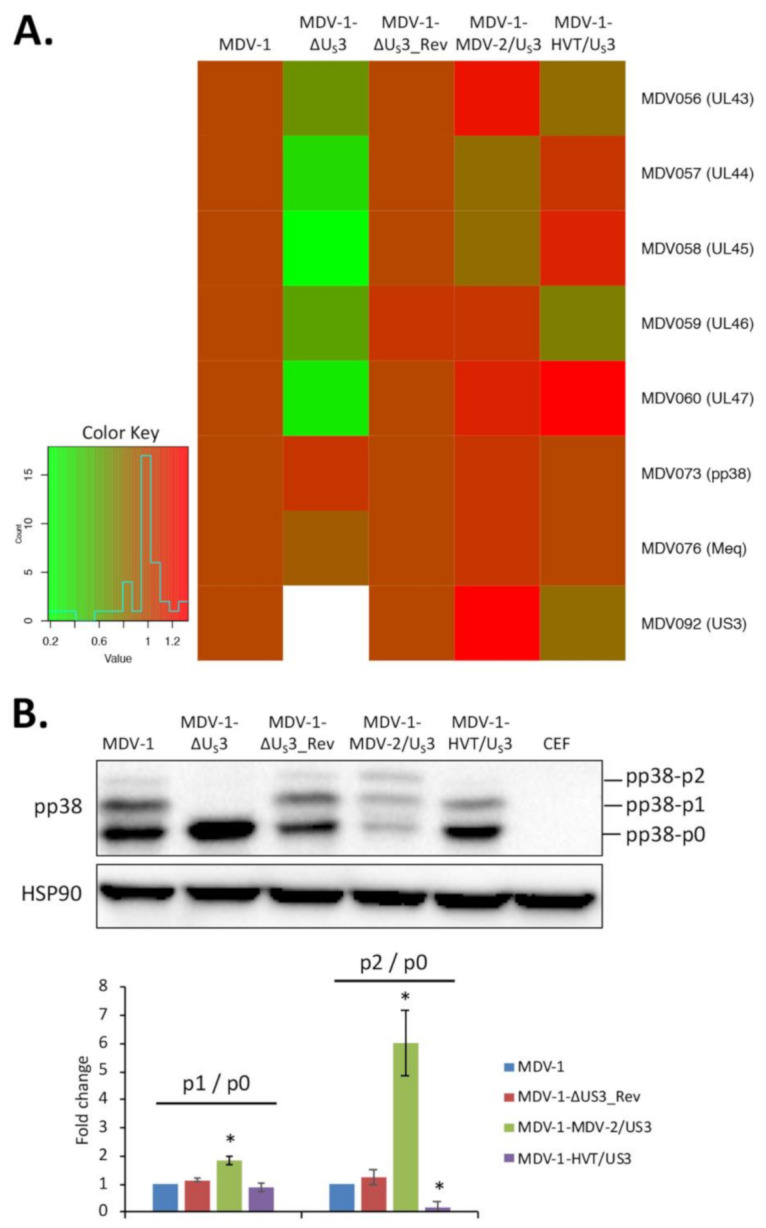
U_S_3 of MDV-1, MDV-2, and HVT differentially regulate viral gene expression. Chicken embryonic fibroblasts (CEF) seeded on 60 mm dishes were infected with 300 plaque-forming units (PFU) of the different viruses or remained uninfected as negative control. Seven days later, cells were harvested for RNA, DNA and protein isolation. (**A**) Gene expression analysis. The extracted genomic DNA was used to determine MDV-1 genome copy number. Total RNA extracted from infected CEF were subjected to cDNA synthesis, followed by quantitative polymerase chain reaction (qPCR) to measure the expression of the indicated MDV-1 genes. The data were analyzed by the 2^−ΔΔCT^ method using chicken *GAPDH* as the internal control and normalized to MDV-1 genome copy number. The relative average fold changes of each gene from two independent experiments are presented as heat map, where red indicates upregulation and green indicates downregulation. (**B**) Analysis of pp38 protein expression. Proteins extracted from infected and uninfected CEF were subjected to sodium dodecyl sulphate-polyacrylamide gel electrophoresis (SDS-PAGE), followed by Western blot analysis with antibodies to MDV pp38 and chicken HSP90 protein, as loading control (upper panel). Phosphorylated pp38 proteins (pp38-p1 and pp38-p2) were quantified with Image J, normalized to unmodified pp38 protein (pp38-p0) and presented as average fold changes, from two independent experiments, of p1 to p0 (bottom graph, left) and p2 to p0 (bottom graph, right) ratios relative to the value derived from parental MDV-1-infected cells. Error bars represent standard deviation (SD). The statistical difference between values derived from parental MDV-1 infected cells and those from other groups was analyzed using a *t*-test. *: *p* < 0.05.

Using chimeric MDVs (Figure 1), we recently reported that U_S_3 from MDV-2 and HVT, close relatives of MDV-1, could partially compensate the function of MDV-1 U_S_3 in virus replication and pathogenesis [17]. In this study, we further determined the role of MDV-1, MDV-2 and HVT U_S_3 in regulating viral gene expression in cell culture and virus replication in lymphoid organs. The construction of U_S_3 deletion (MDV-1-ΔU_S_3), revertant (MDV-1-ΔU_S_3_Rev) and chimeric MDVs (MDV-1-MDV-2/U_S_3, chimeric MDV-1 expressing MDV-2 U_S_3 and MDV-1-HVT/U_S_3, chimeric MDV-1 expressing HVT U_S_3) is briefly outlined in Figure 1, and has been described in detail previously [16,17]. Here, we first analyzed the expression of a cluster of MDV-1 genes (MDV056 to MDV060) encoded by the unique long (U_L_) region of MDV-1, which we previously showed to be down-regulated in MDV-1 U_S_3 null virus-infected chicken embryonic fibroblasts (CEF) [16]. Our results show that, compared to parental MDV-1, the expression of MDV056 to MDV060 was lower in MDV-1-ΔU_S_3-infected CEF, and was fully restored in the revertant virus and differentially compensated by expression of MDV-2 and HVT U_S_3 in chimeric viruses; on the other hand, the expression of *pp38* and *meq* was not affected (Figure 2A). These results suggest that U_S_3 from MDV-1, MDV-2, and HVT differentially regulates viral gene expression. Western blot analysis of pp38 protein, a known substrate of MDV-1 U_S_3, showed that MDV-2 and HVT U_S_3 could compensate the ability of MDV-1 U_S_3 to phosphorylate pp38, resulting in two additional higher molecular weight pp38 protein species (Figure 2B, pp38-p1 and pp38-p2). Interestingly, MDV-2 U_S_3 exhibited a stronger potential to phosphorylate pp38, as the relative amount of phosphorylated pp38 form (Figure 2B, bottom graph), especially the p2 to p0 ratio (p2/p0), in MDV-1-MDV-2/U_S_3-infected cells was significantly greater than that in parental MDV-1-, MDV-1-ΔU_S_3_Rev-, or MDV-1-HVT/U_S_3-infected cells.

**Figure 3:**
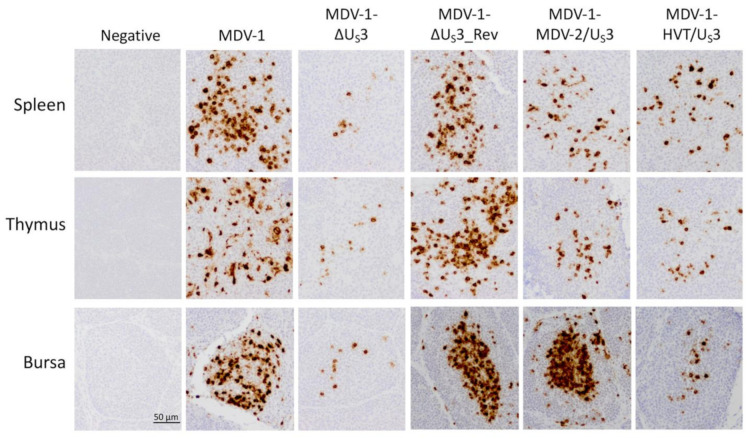
U_S_3 of MDV-1, MDV-2, and HVT differentially regulate virus replication in lymphoid organs. At six days post-inoculation, lymphoid organs (spleen, thymus, and bursa) were collected from virus-inoculated or uninoculated (negative control) chickens, fixed in 10% neutral buffered formalin solution for 48 h, and stored in 70% ethanol until used. Tissue sections were prepared and subjected to immunostaining with anti-pp38 monoclonal antibody and VECTASTAIN ABC kit according to the manufacturer’s instructions, using DAB (3,3’-diaminobenzidine) as the substrate. A representative image of three independent samples from each group is presented; the scale bar is the same for every panel (scale bar = 50 μm).

We have previously shown that deletion of MDV-1 U_S_3 causes the replication deficiency of MDV-1-ΔU_S_3 in splenocytes (determined by virus genome copy number), which could be partially rescued by MDV-2 and HVT U_S_3 [16,17]. In addition, we showed that MDV-2 and HVT U_S_3 may play different roles in the replication of chimeric MDVs in B and T lymphocytes as the inoculation of MDV-1-MDV-2/U_S_3 and MDV-1-HVT/U_S_3 resulted in different levels of bursa and thymus atrophy (Table 1). In this study, we further analyzed the role of U_S_3 from MDV-1, MDV-2, and HVT in regulating virus replication in lymphoid organs using histological methods. Tissue sections prepared from lymphoid organs (spleen, thymus, and bursa), collected from virus-inoculated or uninoculated chickens at day six post-inoculation, were subjected to immunohistochemistry (IHC) analysis of pp38 antigen expression. Our results show that there was a smaller number of cells expressing pp38 in the spleen of MDV-1-ΔU_S_3-inoculated chickens compared to parental and revertant viruses, which was partially restored in MDV-1-MDV-2/U_S_3 and MDV-1-HVT/U_S_3-inoculated chickens (Figure 3, spleen). Similar results were observed in the thymus, where MDV-2 and HVT U_S_3 partially increased the number of pp38-expressing cells (Figure 3, thymus). Interestingly, the pp38 expression pattern in the bursa of chickens inoculated with MDV-1-MDV-2/U_S_3 was similar to parental and revertant MDV-1 and there were clearly more cells expressing pp38 than in the bursa of MDV-1-ΔU_S_3- and MDV-1-HVT/U_S_3-inoculated chickens (Figure 3, bursa). These bursa and thymus histological results are consistent with our previous report that infection with MDV-1-MDV-2/U_S_3 resulted in bursa atrophy comparable to parental and revertant viruses, while inoculation of MDV-1-ΔU_S_3 and MDV-1-HVT/U_S_3 did not induce bursa atrophy, and inoculation of MDV-1-ΔU_S_3, MDV-1-MDV-2/U_S_3 and MDV-1-HVT/U_S_3 all resulted in a similar level of mild thymus atrophy (Table 1). Taken together, these results suggest that U_S_3 from MDV-1, MDV-2, and HVT may play different roles in the replication of MDV-1 in B and T lymphocytes.

**Figure 4:**
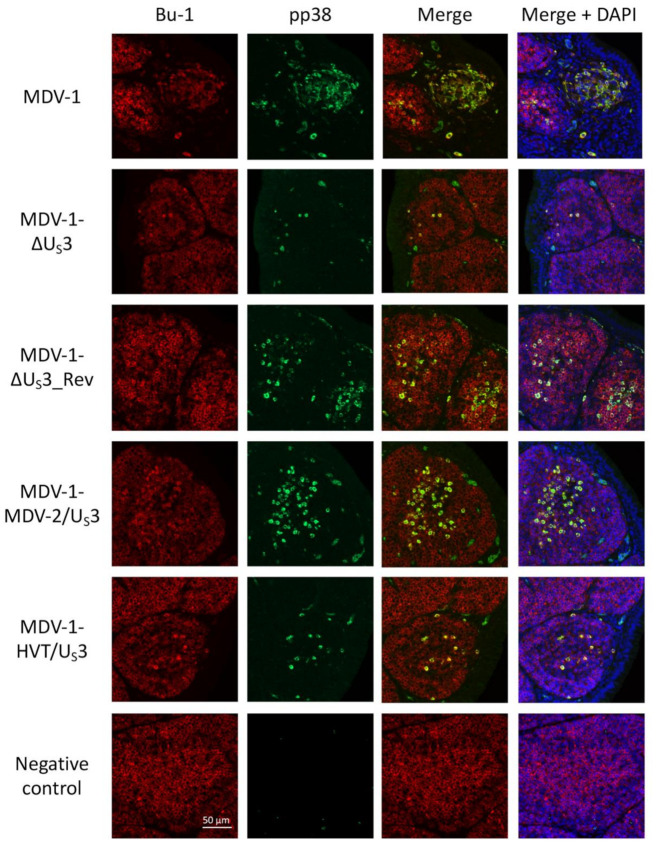
U_S_3 of MDV-1, MDV-2, and HVT differentially regulate virus replication in bursal B lymphocytes. Bursa tissue sections, prepared from virus-inoculated or uninoculated (negative control) chickens, were subjected to immunofluorescence assay. After blocking, sections were incubated with mouse anti-pp38 antibody for 1 h at room temperature, followed by another hour incubation with Alexa Flour 488 conjugated goat anti-mouse antibody. After three washes with phosphate-buffered saline (PBS), sections were stained with Alexa Flour 647 conjugated mouse anti-chicken Bu-1 antibody (B lymphocyte marker) for 1 h at room temperature. 4′,6-diamidino-2-phenylindole (DAPI) was used to stain cell nuclei. A representative image of three independent samples from each group is presented; the scale bar is the same for every panel (scale bar = 50 μm).

To specifically study the role of U_S_3 from MDV-1, MDV-2, and HVT in regulating virus replication in B lymphocytes, we performed sequential dual immunofluorescence assay (IFA) using Bu-1, a B lymphocyte marker, and pp38, an MDV marker, in bursa sections collected from virus-inoculated or uninoculated chickens at day six post-inoculation. Since only a mouse anti-chicken Bu-1 monoclonal antibody was available, bursa sections were incubated sequentially with mouse anti-pp38 monoclonal antibody, Alexa Flour 488 conjugated goat anti-mouse antibody, and Alexa Flour 647 conjugated mouse anti-chicken Bu-1 monoclonal antibody each for 1 h at room temperature, with three phosphate-buffered saline (PBS) washes after incubation with each antibody. The merged IFA image shows that the majority of pp38 expressing cells are B lymphocytes located in the bursa medulla of chickens inoculated with parental MDV-1 (Figure 4, MDV-1). Consistent with the IHC results in Figure 3, a similar pattern of B lymphocytes expressing pp38 was observed in the bursa of parental, revertant and MDV-1-MDV-2/U_S_3-inoculated chickens, and there were more pp38-expressing B lymphocytes than in the bursa of MDV-1-ΔU_S_3- and MDV-1-HVT/U_S_3-inoculated chickens (Figure 4). These results suggest that MDV-2 U_S_3, but not HVT U_S_3, could fully compensate the role of MDV-1 U_S_3 in regulating the replication of MDV-1 in B lymphocytes. Overall, this study demonstrates that U_S_3 from MDV-1, MDV-2 and HVT differentially regulate virus replication in lymphoid organs.

Using an MDV quantitative polymerase chain reaction (qPCR) array, we previously determined that 34 MDV-1 genes are differentially regulated by deleting MDV-1 U_S_3 [16]. Out of these gene, we noticed that a cluster of U_L_ genes, including MDV056 (UL43, probable membrane protein), MDV057 (UL44, virion membrane glycoprotein C), MDV058 (UL45, envelope/membrane protein), MDV059 (UL46, tegument phosphoprotein) and MDV060 (UL47, tegument phosphoprotein), are highly down-regulated in MDV-1 U_S_3 null virus [16]. We and others have shown that MDV-1 U_S_3 is important for MDV-1 genome replication and plaque-forming efficacy [12,13,16], and that MDV-2 and HVT U_S_3 could partially compensate the role of MDV-1 U_S_3 in these processes [17]. The downregulation of genes encoding membrane or tegument proteins (e.g., MDV056 to MDV060) may be one of the reasons contributing to the growth deficiency of MDV-1 U_S_3 null virus. Our results here show that the expression of MDV056 to MDV060 was differentially compensated in CEF infected with MDV-1-MDV-2/U_S_3 and MDV-1-HVT/U_S_3 (Figure 2A), which could explain, at least partially, the role of MDV-2 and HVT U_S_3 in rescuing the growth deficiency of MDV-1 U_S_3 null virus in vitro. Furthermore, our IHC results show that the smaller number of cells expressing pp38 in the spleen and thymus of chickens inoculated with MDV-1-ΔU_S_3 was partially rescued by MDV-2 and HVT U_S_3, and MDV-2 U_S_3, but not HVT U_S_3, could fully rescue the number of cells expressing pp38 in the bursa (Figure 3). These results provide the histological evidence for our previous report on genome copy number [17] and lymphoid organ atrophy induced by the inoculation of MDV-1-MDV-2/U_S_3 and MDV-1-HVT/U_S_3 (Table 1). Notably, for the first time, we demonstrate that MDV-1 U_S_3 is important for the replication of MDV-1 in B lymphocytes using dual IFA; in particular, MDV-2 U_S_3, but not HVT U_S_3, could fully compensate MDV-1 U_S_3 to rescue the replication deficiency of MDV-1 U_S_3 null virus in B lymphocytes (Figure 4), even though both MDV-2 and HVT U_S_3 exhibit a similar level (59% and 60%, respectively) of amino acid sequence identity to MDV-1 U_S_3 (Figure S1). In addition, by comparing the amino acid sequence, we noticed that ~100 amino acids at the amino-terminus of U_S_3 from MDV-1, MDV-2, and HVT are highly variable (Figure S1), which might contribute the differential functions observed for U_S_3 from MDV-1, MDV-2, and HVT. Another interesting result we noticed is that both MDV-2 and HVT U_S_3 could fully compensate the replication of MDV-1 U_S_3 null virus in feather follicle epithelium (FFE) [17], suggesting that the replication of MDV-1 in lymphocytes and the epithelium is regulated differently. Even though the mechanisms involved remain to be explored, these results emphasize the diverse functions of U_S_3 from MDV-1, MDV-2, and HVT. The mechanisms of MDV U_S_3 regulation of virus replication and gene expression will be the focus of future studies.

Recent studies have challenged the widely accepted dogma of MDV-1 infection in immune cells [3]. The limited availability of commercial antibodies for chicken immune cell markers restricts the study of the interplay between MDV-1 infection and the host immune system. The development of more antibodies specific for chicken cell markers and the use of sequential dual IFA strategies described here and elsewhere opens up the possibility to precisely analyzing MDV-1 infection in specific cell types, which would help elucidate the life cycle of MDV-1 in chickens.

## Figures and Tables

**Table 1:** Lymphoid organs to body weight ratio of inoculated and negative control chickens on day 14 post-inoculation.

Virus	Bursa/Body Weight × 100	Thymus/Body Weight × 100
Negative	0.422 ± 0.100 ^a^	0.741 ± 0.098 ^a^
MDV-1	0.213 ± 0.079 ^b^	0.283 ± 0.113 ^c^
MDV-1-ΔU_S_3	0.419 ± 0.057 ^a^	0.495 ± 0.106 ^b^
MDV-1-ΔU_S_3_Rev	0.141 ± 0.072 ^b^	0.204 ± 0.050 ^c^
MDV-1-MDV-2/U_S_3	0.188 ± 0.078 ^b^	0.426 ± 0.152 ^b^
MDV-1-HVT/U_S_3	0.391 ± 0.084 ^a^	0.429 ± 0.091 ^b^

On day 14 post-inoculation, five chickens from each group were euthanized and the weights of their lymphoid organs (bursa and thymus) and bodies were measured. Data were presented as a ratio of the bursa or thymus to body weight multiplied by 100. The value in the table represents the average ratio derived from five chickens ± standard deviation. Indices with different letter superscripts are statistically different (*p* < 0.05). Table 1 is adapted from Figure 2B of “Marek’s disease virus U_S_3 protein kinase phosphorylates chicken HDAC 1 and 2 and regulates viral replication and pathogenesis” by Liao Y, Lupiani B, AI-Mahmood M, Reddy SM (2021). PLoS Pathog 17(2): e1009307. Copyright 2021 by Liao et al.

## Data Availability

Data is contained within the article or supplementary material.

## Supplementary Materials

The following is available online at https://www.mdpi.com/article/10.3390/microorganisms9040785/s1, Figure S1: Multiple sequence alignment of alphaherpesvirus U_S_3 protein kinase.
