# Supplementary material for: US3 Serine/Threonine Protein Kinase from MDV-1, MDV-2, and HVT Differentially Regulate Viral Gene Expression and Replication
Source: Microorganisms. 2021 Apr 9;9(4):785. doi: 10.3390/microorganisms9040785 (PMC8069862; doi:10.3390/microorganisms9040785)
Supplement: Supplementary file 1 [file microorganisms-09-00785-s001.pdf]

Supplementary figure

A.

```
MDV-1      ------------------------------------------------------------ 0
MDV-2      ------------------------------------------------------------ 0
HVT        ------------------------------------------------------------ 0
HSV-1      MACRKFCRVYGGQGRRKEEAVPPETKPSRVFPHGPFYTPAEDACLDSPPPETPKPSHTTP 60
VZV        ------------------------------------------------------------ 0


MDV-1      ------------------------MSSSPEAETMECGISSSKVHDSKTNTTY-------G 29
MDV-2      ---------------------------------METNELSSKVSDYNANRPY-------E 20
HVT        ---------------------------------MEVDVESSKVSAS----NM-------G 16
HSV-1      PGDAERLCHLQEILAQMYGNQDYPIEDDPSADAADDVDE-DAPDDVAYPEEYAEELFLPG 119
VZV        ---------------------------------MNDV---------DATDTF----VGQG 14
                                             :

MDV-1      IIHNSINGTDT-----------TL-F----DTFPDSTDNAEVTGDVDDVKTESSPESQSE 73
MDV-2      TIRSDTS---------------DTDPSVSCGTLSDKDGDDEESIDLSKVPNA-TNVGAGE 64
HVT        IVCENIESGTT-----------VAEPSMSPDTSNNSFDNEDFRGPEYDVEINTRKSANLD 65
HSV-1      DATGPLIGANDHIPPPRG--------ASPPGIRRRSRDEIGATG----FTAEE-----LD 162
VZV        KFRGAISTSPSHIMQTCGFIQQMFPVEMSPGIESEDDPNYD-VN----MDIQS-----FN 64
                                         .    .  :         .          :

MDV-1      DLSPFGNDGNESPETVTDIDAVSAVRMQYNIVSSLSPGSEGYIYVCTKRGDNTKRKVIVK 133
MDV-2      DCTSPNDGRTELCRTTSVTGPASVVRMQYNIISPLPPSSEGRVFVCTRWDDVSNKKVIVK 124
HVT        RMESSCREQRAACELRKCSCPTSAVRMQYSILSSLAPGSEGHVYICTRYGDADQKKCIVK 125
HSV-1      AMDRQAARAISRGGKPPSTMAKLVTGMGFTIHGALTPGSEGCVFDSSHPDY--PQRVIVK 220
VZV        IFDGVHETEAEASVALCAEARVGINKAGFVILKTFTPGAEGFAFACMDSKT--CEHVVIK 122
                                       : *   : *.:**  : .        .: ::*

MDV-1      AVTGGKTLGSEIDILKKMSHRSIIRLVHAYRWKSTVCMVMPKYKCDLFTYIDIM-GPLPL 192
MDV-2      VVTGGRDPGREIEIVKTLSHCAIIQLIHAYSWKSTVCMVMPKYKCDLFTYVDRK-ESIPL 183
HVT        AVVGGKNPGREVDILKTISHKSIIKLIHAYKWKNVVCMAMRVYRYDLFTYIDGV-GPMPL 184
HSV-1      AGW-YTSTSHEARLLRRLDHPAILPLLDLHVVSGVTCLVLPKYQADLYTYLSRRLNPLGR 279
VZV        AGQ-RQGTATEATVLRALTHPSVVQLKGTFTYNKMTCLILPRYRTDLYCYLAAKRN-LPI 180
           .       . *  ::: : * ::: *   .  .  .*: :  *: **: *:      :

MDV-1      NQIITIERGLLGALAYIHEKGIIHRDVKTENIFLDKPENVVLGDFGAACKLDEHTDKPKC 252
MDV-2      KDVIVIERRLLEALVYLHGKGVIHRDVKTENIFLDYPGNAVLGDFGAACKLDMHDNSPKC 243
HVT        QQMIYIQRGLLEALAYIHERGIIHRDVKTENIFLDNHENAVLGDFGAACQLGDCIDTPQC 244
HSV-1      PQIAAVSRQLLSAVDYIHRQGIIHRDIKTENIFINTPEDICLGDFGAACFVQGSRSSPFP 339
VZV        CDILAIQRSVLRALQYLHNNSIIHRDIKSENIFINHPGDVCVGDFGAACFPVDINAN-RY 239
            ::  :.* :* *: *:* ..:****:*:****::   :  :*******       .

MDV-1      YGWSGTLETNSPELLALDPYCTKTDIWSAGLVLFEMSVKNITFFGKQV----NGSGSQLR 308
MDV-2      YGWAGTMETNSPELLALDPYCAKTDIWSAGLVLFEMSAKKRTLFGKQV----KTSSSQLR 299
HVT        YGWSGTVETNSPELSALDPYCTKTDIWSAGLVLYEMAIKNVPLFSKQV----KSSGSQLR 300
HSV-1      YGIAGTIDTNAPEVLAGDPYTTTVDIWSAGLVIFETAVHNASLFSAPRGPKRGPCDSQIT 399
VZV        YGWAGTIATNSPELLARDPYGPAVDIWSAGIVLFEMATGQNSLFERDGLDGNCDSERQIK 299
           ** :**: **:**: * ***   .******:*::* :  :  :*          .  *:

MDV-1      SIIRCLQVHPLEFPQNNSTNLCKHFKQYAI-QLRHPYAIPQIIRKSGMTMDLEYAIAKML 367
MDV-2      ALIRCLQIHALEFPQDESTTLCKQFKQYAI-PLRPPFSIPEVVRRNIPSMDVEYTIAKML 358
HVT        SIIRCMQVHELEFPRNDSTNLCKHFKQYAV-RVRPPYTIPRVIRNGGMPMDVEYVISKML 359
HSV-1      RIIRQAQVHVDEFSPHPESRLTSRYRSRAAGNNRPPYTRPAWTRYYKMDIDVEYLVCKAL 459
VZV        LIIRRSGTHPNEFPINPTSNLRRQYIGLAKRSSRKPGSRPLWTNLYELPIDLEYLICKML 359
            :**    *  **  .  : *  ::   *    * * : *   .     :*:** :.* *

MDV-1      TFDQEFRPSAQDILMLPLFTKEPADALYTITAAHM  402
MDV-2      TFDQEFRPSAQDILAFPLFVKEAPQNLQALFVP--  391
HVT        TFDQEFRPSAKEILNMPLFTKAPINLLNITPSDSV  394
HSV-1      TFDGALRPSAAELLCLPLFQQK-------------  481
VZV        SFDARHRPSAEVLLNHSVFQTLPDPYPNPMEVGD-  393
           :**   ****  :*   :*
```

B.

	MDV-1	MDV-2	HVT	HSV-1	VZV
**MDV-1**	100				
**MDV-2**	59	100			
**HVT**	60	53	100		
**HSV-1**	36	33	36	100	
**VZV**	32	32	33	39	100

Figure S1. Comparative analysis of U_S3 from alphaherpesviruses. (A) Amino acid sequence alignment of U_S3 from MDV-1, MDV-2, HVT, herpes simplex virus type 1 (HSV-1), and varicella-zoster virus (VZV). The ATP binding site and catalytic active site are highlighted in yellow and green, respectively. (B) The amino acid percent identify matrix of U_S3 from MDV-1, MDV-2, HVT, HSV-1, and VZV.
